# An Ensemble of CNN Models for Parkinson’s Disease Detection Using DaTscan Images

**DOI:** 10.3390/diagnostics12051173

**Published:** 2022-05-08

**Authors:** Ankit Kurmi, Shreya Biswas, Shibaprasad Sen, Aleksandr Sinitca, Dmitrii Kaplun, Ram Sarkar

**Affiliations:** 1 Department of Computer Science and Engineering, Kalyani Government Engineering College, Kalyani 741235, West Bengal, India; ankitkurmi152@gmail.com; 2 Department of Electronics and Telecommunication Engineering, Jadavpur University, Kolkata 700032, West Bengal, India; mimigg443@gmail.com; 3 Department of Computer Science and Technology, University of Engineering and Management, Kolkata 700160, West Bengal, India; shibubiet@gmail.com; 4 Research Centre for Digital Telecommunication Technologies, Saint Petersburg Electrotechnical University ”LETI”, 197022 St. Petersburg, Russia; amsinitca@etu.ru; 5 Department of Automation and Control Processes, Saint Petersburg Electrotechnical University ”LETI”, 197022 St. Petersburg, Russia; dikaplun@etu.ru; 6 Department of Computer Science and Engineering, Jadavpur University, Kolkata 700032, West Bengal, India

**Keywords:** Parkinson’s disease, CNN Model, ensemble method, DaTscan images

## Abstract

Parkinson’s Disease (PD) is a progressive central nervous system disorder that is caused due to the neural degeneration mainly in the substantia nigra in the brain. It is responsible for the decline of various motor functions due to the loss of dopamine-producing neurons. Tremors in hands is usually the initial symptom, followed by rigidity, bradykinesia, postural instability, and impaired balance. Proper diagnosis and preventive treatment can help patients improve their quality of life. We have proposed an ensemble of Deep Learning (DL) models to predict Parkinson’s using DaTscan images. Initially, we have used four DL models, namely, VGG16, ResNet50, Inception-V3, and Xception, to classify Parkinson’s disease. In the next stage, we have applied a Fuzzy Fusion logic-based ensemble approach to enhance the overall result of the classification model. The proposed model is assessed on a publicly available database provided by the Parkinson’s Progression Markers Initiative (PPMI). The achieved recognition accuracy, Precision, Sensitivity, Specificity, F1-score from the proposed model are 98.45%, 98.84%, 98.84%, 97.67%, and 98.84%, respectively which are higher than the individual model. We have also developed a Graphical User Interface (GUI)-based software tool for public use that instantly detects all classes using Magnetic Resonance Imaging (MRI) with reasonable accuracy. The proposed method offers better performance compared to other state-of-the-art methods in detecting PD. The developed GUI-based software tool can play a significant role in detecting the disease in real-time.

## 1. Introduction

Parkinson’s disease (PD) has a prevalence rate of 1% in the over-60 age group, and affects about 0–2 per 1000 people. It is the second most common brain disease after Alzheimer’s disease [1]. A central nervous system disorder, especially those affecting the brain, causes the neurons to degenerate. A person suffering from this disease will experience tremors at rest, bradykinesia (slow movement), rigidity, sleep disturbances, asymmetry in posture, depression, and other such symptoms. In the advanced stages of the disease, PD dementia becomes coarse and patients have difficulty sleeping or concentrating. People with PD lose the nerve endings that produce dopamine, the prime chemical which controls most of the involuntary functions of the body. This might help explain some of the involuntary symptoms of PD, like tiredness, non-uniform blood pressure, reduced peristalsis, and a sudden drop in blood pressure.

PD appears hereditary in some cases, and certain mutations can be traced to it, but most of the time this disease is random. There is a growing consensus that it is caused by a combination of genetics and environmental factors, such as exposure to toxins. A loss of dopaminergic neurons in the substantia nigra region of the brain is one of the leading causes of Parkinson’s disease [2]. Currently, there is no particular test for its diagnosis [3]. The diagnosis till date is primarily based on the symptoms mentioned above and their response to PD medications. However, non-invasive imaging like Positron Emission Tomography (PET) scans can help with the diagnosis. Since these are not purely scientific, the need for Artificial Intelligence (AI) based techniques for diagnosis become important. Researchers have been addressing the need for AI-based systems because many of them have been so far adopted successfully in different medical imaging applications [4,5,6].

The aim of this paper is to present an ensemble approach for the detection of PD that integrates decision scores obtained from four different DL models. In addition to assisting practitioners in performing disease diagnosis, the outcomes of this model will enable physicians to take action before patients’ disorders become more serious. The present study has been conducted using a publicly available database of DaTscan Single Photon Emission Computerized Tomography (SPECT) images accessed from the PPMI data [7]. The proposed model provides a higher recognition score than many of the existing methodologies in the literature.

The organization of the paper is as follows: some co-related works for the classification of PD have been mentioned in Section 2. Section 3 describes the motivation and overview of the proposed work. The details regarding the dataset used, along with the pre-processing steps applied to the dataset have been mentioned in Section 4. In Section 5, we have explained the methodology used in the present experiment including the details of the base models and the applied ensemble approach. Section 6 describes the results obtained by the proposed model and also compares its performance with other state-of-the-art techniques found in the literature. Section 7 describes the application that is developed by using the proposed methodology. Finally, Section 8 and Section 9 discuss and conclude the overall work.

## 2. Related Work

Till date, researchers across the world have been trying to observe the outcomes of various Machine Learning (ML) and DL-based methods for prediction of PD. Though several of these techniques have provided satisfactory results, it has also been noticed that different models yield different outcomes.

This section briefly highlights a few of the approaches available in the literature. Abos et al. [8] extracted features from Resting-State Functional MRI (rsfMRI) and used Support Vector Machine (SVM) for the detection of PD. They achieved an 86.96% accuracy, 78.95% sensitivity, and a specificity of 92.59%. Amoroso et al. [9] used network and clinical features to classify PD patients using an SVM. They experimented on the PPMI dataset and got a 93% recognition accuracy and sensitivity, and 92% of specificity. A Sparse feature selection model was proposed by Lei et al. [10], reporting an accuracy of around 80%. Salvatore et al. [11] considered healthy, PD, and supranuclear palsy MRI images to extract features. Next, they have used Principal Components Analysis (PCA) to find the relevant features and fed them to an SVM classifier for classification purposes, having obtained above 90% accuracy for the case of PD patients vs. controls. Prashant et al. [12] used SVM with striatal binding ratio to classify PD patients and they got an accuracy of 96.14%, a sensitivity score of 95.74%, and 77.35% specificity.

Brahim et al. [13] performed their experiments for classifying PD using shape and surface-fitting-based features and an SVM classifier. They achieved a 92.6% accuracy, a 91.2% sensitivity, and a specificity of 93.1%. An Artificial Neural Network (ANN) architecture for PD classification was proposed by Rumman et al. [14] and they obtained an accuracy of 94%, sensitivity of 100%, and specificity of 88%.

Sivaranjani et al. [15] proposed a Convolutional Neural Network (CNN) trained on the PPMI dataset and achieved an accuracy of 88.9%. Another DL-based framework was proposed by Esmaeilzadeh et al. [16] for classification and regression of PD on PPMI images. Shah et al. [17] have shown the effectiveness of their proposed CNN-based model used for the categorization of PD on the PPMI MRI dataset with good results. Another work to detect PD from Neuromelanin sensitive MRI using a CNN has been shown in [18] that has achieved an 85% accuracy. Magesh et al. [19] trained the VGG16 model on the PPMI dataset and obtained a 95.2% accuracy, and a specificity of 90.9%. Quan et al. [20] considered the transfer learning concept and used the InceptionV3 model in their experiment of predicting PD. They obtained a 98.4% accuracy, a sensitivity score of 98.8%, and a specificity score of 97.6%. Whereas, Ortiz et al. [21] trained two DL models—AlexNet and LeNet for the classification of PD and Health Control. They achieved a better accuracy of 95 ± 0.3% when using AlexNet.

After analyzing the methods reported in [18,19,20,21], we have observed that most of the models have some limitations. For example, a few models [17,19] have shown higher false positive rate, whereas some others [18,21] have shown higher false negative rate. The probable reason for that may be the weakness of the models to deal with the nature of data. According to the authors in [19], this may happen due to abnormal increase in dopamine activity in the Region of Interest (ROI) of the scans.

On the other hand, the literature reveals that the fusion techniques have already been applied successfully in distinct domains to produce a better result than any individual learning model [22,23,24]. An ensemble is a model which is used to combine the predictions made by different learning models. The predictions made by the members of an ensemble model may be combined using statistics (like mode or mean) or they can be combined using more sophisticated strategies. Generally, an ensemble model tries to learn how much to rely on each member and under what conditions. Though ensemble methods come with additional computational cost and complexity, there are reasons to use an ensemble model. Usually, an ensemble model makes better predictions and shows superior performance over a single learning model. Also, such a model reduces the dispersion of the predictions of the different base models. From the literature it can be observed that ensemble techniques have shown competent results in varied domains like predicting COVID-19 using CT scans [25], human activity recognition using sensor data [26], breast cancer detection using histopathology images [27], plant identification using leaf images [28], cervical cancer detection [29], handwritten music symbol recognition [30].

However, a limited number of research works are there which try to improve the overall classification accuracy of PD by introducing ensemble-based techniques applied to ML approaches. The author in [31] has shown the usefulness of the K-Nearest Neighbours (KNN) ensemble technique for the detection of PD. Authors in [32] combined SVM with linear kernel classifiers for different tests considering RNA, Cerebrospinal Fluid, Serum tests, and pre-processed neuro-images features from PPMI database subjects. Table 1 highlights a few past methods proposed so far in this domain.

## 3. Motivation and Overview

As in most cases, it has been observed that DL models perform better as compared to ML models due to their ability to extract powerful features automatically from inputs using convolution and pooling operations. Hence, in this work, we have considered DL models as the base learners. It is to be noted that DL based neural networks are actually nonlinear networks which come with better flexibility and also scale in proportion to the training data available. However, a flip side of this flexibility is that these models generally learn through a stochastic training method, and due to this they become very sensitive to the training data. Also, they may find a varied set of weights every time the models are trained, and hence they generate varied predictions about the input samples. A competent alternative to minimize the variance of neural network models can be to use different models instead of a single model, and to unite the prediction scores obtained from these models.

Keeping this fact in mind, we have used an ensemble learning approach where different standard CNN models are used to generate the initial predictions from the input DaTscan images related to PD, which are then combined using a Fuzzy-ranked based fusion approach. Although literature of PD detection divulges that a few number of researchers have made an attempt to apply ensemble approaches which are very naive, and hence may fail to capture information yielded by different learning models intelligently.

## 4. Dataset

The present experiments were conducted using a dataset containing 645 DaTscan SPECT images extracted from the Parkinson’s Progression Markers Initiative (PPMI) [7] DaTscan images were widely used in the automatic diagnosis of Parkinson’s Disease after being preprocessed and reorganized from PPMI SPECT images. Each PPMI SPECT image, then, is built into a volume of 91 × 109 × 91 [37,38].

### 4.1. Dataset Preparation

**Figure 1:**
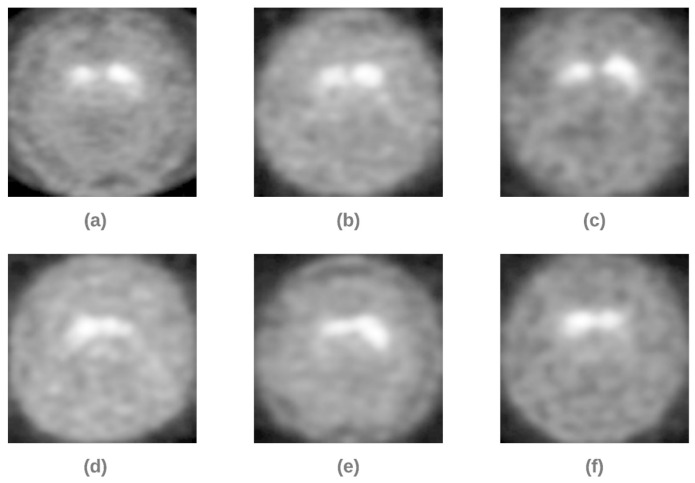
Sample images (**a**–**f**) from PPMI dataset for a person suffering from PD.

**Figure 2:**
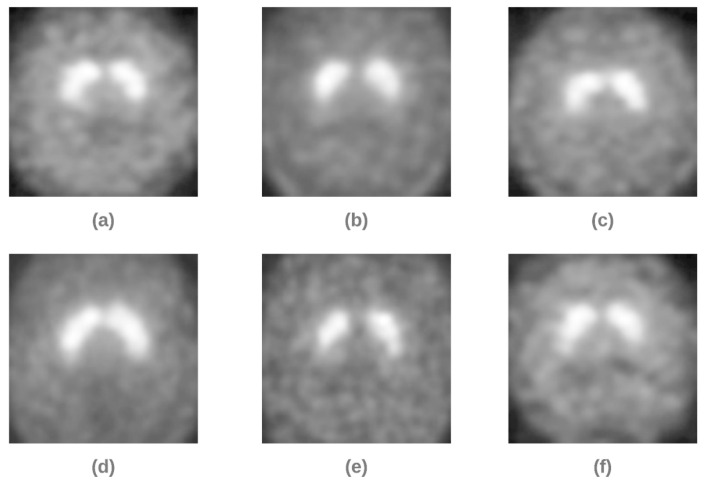
Sample images (**a**–**f**) from PPMI dataset for a person not suffering from PD.

All the DaTscan images were in DICOM format, and each consisted of 91 slides of shape 109 × 91. To make them fit for the current study, we extracted the 41’st slide from every DaTscan image and converted it into png format. Due to the difference in the size of the brain of males and females, we cropped the extra unnecessary black portion. This resulted in the irregularity of the dimensions of the extracted images. To fit the extracted images into our DL models, we resized them to 224 × 224 resolution and were scaled between [0, 1], keeping the brightness range between [0.1, 1.5]. Figure 1 shows some of the sample images from the PPMI dataset for a person having PD and Figure 2 shows samples from PPMI dataset for a person without PD.

### 4.2. Dataset Splitting

The dataset, consisting of a total of 645 images (432 PD and 213 non-PD), is randomly divided into an 80:20 ratio for train-test splitting. The details of the images (PD and non-PD) present in the train and test sets have been mentioned in Table 2.

## 5. Proposed Methodology

**Figure 3:**
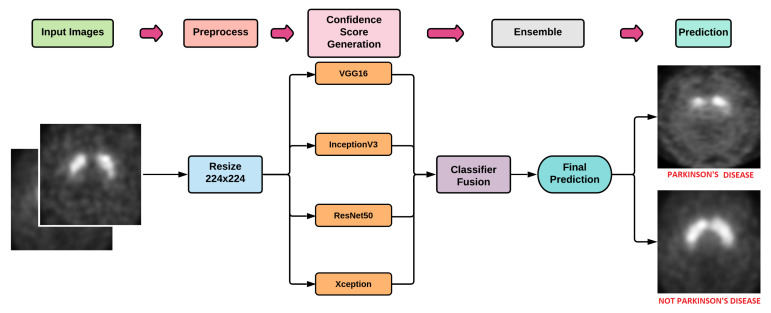
Workflow of the proposed work.

In the current work, initially we have trained four popularly used DL models namely VGG16 [39], Xception [40], ResNet50 [41], and Inception-V3 [42] on the training set of PPMI dataset. The trained models have been used for the evaluation of the test set. The obtained outcomes from these four models are then ensembled using the Fuzzy Rank Level Fusion (FRLF) based approach to elevate the overall performance of the model. Figure 3 shows the basic workflow of the proposed work.

### 5.1. DL Models

In the current work, we have used VGG16, ResNet50, Inception-V3, and Xception models to train the training dataset. A brief description of the four DL models is mentioned in the following subsections.

#### 5.1.1. VGG16

VGG16 was one of the best performing architectures in the ImageNet Large Scale Visual Recognition Challenge (ILSVRC) 2014 [39]. The model achieved a 92.7% test accuracy on the ImageNet dataset. The model contains a total of 16 layers - 13 Convolutional layers, 3 Fully Connected layers, 5 Max Pooling layers, and a Softmax layer.

**Figure 4:**
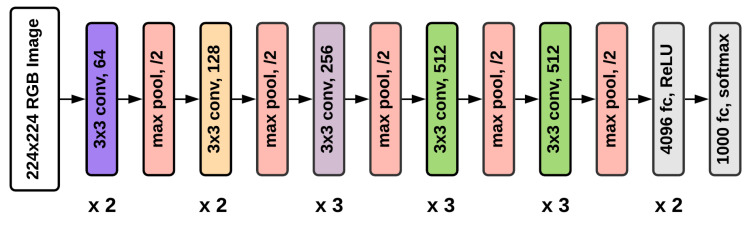
Architecture of VGG16 model.

All the hidden layers in this model use Rectified Linear Unit (ReLU) as its activation function. ReLU results in faster learning and also decreases the likelihood of vanishing gradient. To solve the current binary classification problem, we have added a final layer of Softmax activation. Figure 4 depicts the VGG16 architecture.

#### 5.1.2. ResNet50

**Figure 5:**
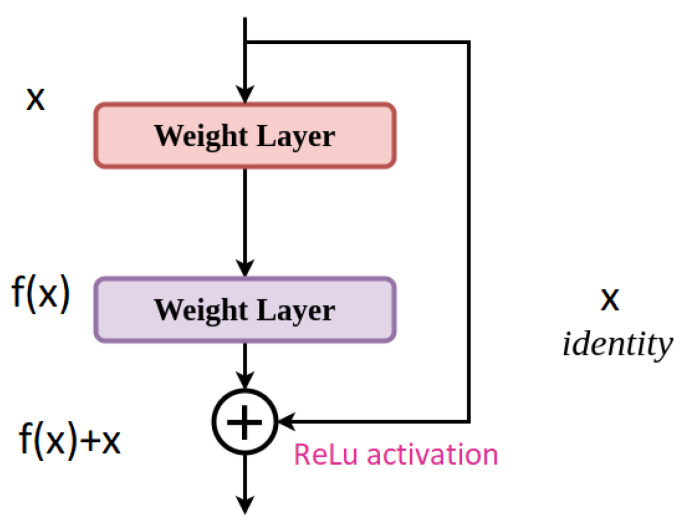
Architecture of ResNet50 model.

ResNet50 [41] introduces a 50-layer deep residual learning framework having shortcut connections that simply perform identity mappings. We have added a final layer of Softmax activation for the binary classification problem under consideration. Figure 5 depicts the architecture of the ResNet50 model.

#### 5.1.3. Inception-V3

**Figure 6:**
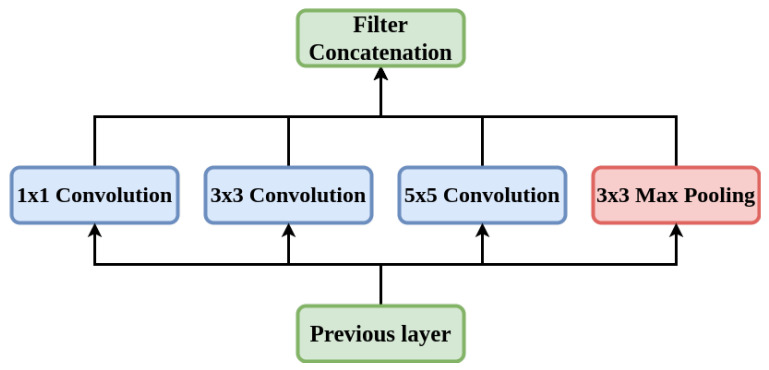
Architecture of Inception-V3 model.

Inception-V3 [42] is a frequently used model for the image classification tasks. This model is composed of symmetric and asymmetric constituents including layers like convolution, average and max pooling, and fully connected layers. Despite having a 42-layer architecture and approximately 12 million parameters, the cost of computation is remarkably less and it is very much efficient than VGGNet [39]. Figure 6 depicts the architecture of the Inception-V3 model.

#### 5.1.4. Xception

Xception stands for “extreme inception”. It re-frames the way we look at neural nets—Convolution Nets in particular. As the name suggests, it takes the principle of Inception to an extreme. In Xception there is no intermediate activation function for non-linearity [40].

**Figure 7:**
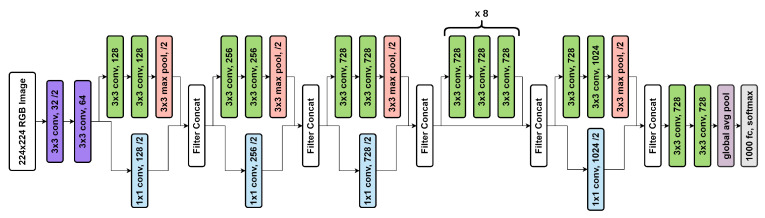
Architecture of the Xception model.

We have added a final layer of Softmax activation for the current binary classification problem. The architecture is shown in Figure 7.

In this work, we have trained VGG16, ResNet50, Inception-V3, and Xception models over a total of 500 epochs with batch size and step size of 16 and 32, respectively for each of the epochs. The learning rate of all the models was set to 0.001, and Adam optimizer has been used to handle sparse gradients on noisy images [43]. Confidence scores of these base models are then ensembled to improve the overall performance. The ensembled method used here is detailed in the next subsection.

### 5.2. Ensemble Method

Essentially, classifier combinations are developed from the idea that each classifier operates in a unique way so that different outcomes can be observed depending on the classifier. So, choosing only one classifier might not be the best idea since the used classifier might not be able to extract potential useful information. In order to avoid this problem, ensemble methods can be used that take into account the outcomes of the various classifiers and make the final choice so that overall accuracy is enhanced. The FRLF [44] algorithm here generates fuzzy ranks by using the confidence scores of a classifier on a Gaussian function. It compares the proximity of classifier outputs as opposed to conventional ranking methods. When a return is ideal, the fuzzy rank is 0, which corresponds to the highest rank (rank 1) in conventional ranking; when the outcome is far away from the ideal, the fuzzy rank gradually approaches unity. This ensemble approach aims to generate a ranking system based on the confidence scores of the base learners, which will become apparent later in this section.

The FRLF method can be expressed mathematically as follows. Let there be N different models ($M_{1}$, $M_{2}$, …, $M_{N}$) for a particular input. In our case, the value of N is 4, as mentioned above.

In the first step, our proposed system chooses a model (say $M_{1}$) and generates confidence scores for all the corresponding classes. Let the confidence scores be ($CS_{1}^{M_{1}}$, $CS_{2}^{M_{1}}$, …, $CS_{C}^{M_{1}}$). The confidence scores are then used to calculate fuzzy ranks. The Gaussian density function is used to assign lesser rank to scores with higher confidence. Let the fuzzy ranks be ($R_{1}^{M_{1}}$, $R_{2}^{M_{1}}$, …, $R_{C}^{M_{1}}$) where C represents the sum of the number of distinct classes in consideration. In specific, The $CS_{i}^{M_{1}}$ and $R_{i}^{M_{1}}$ represent the confidence score and fuzzy rank of the ith class while classifying through the model $M_{1}$. Correspondingly, we have ($CS_{1}^{M_{2}}$, $CS_{2}^{M_{2}}$, …, $CS_{C}^{M_{2}}$) and ($R_{1}^{M_{2}}$, $R_{2}^{M_{2}}$, …, $R_{C}^{M_{2}}$) and so on for the models used in the experiments.

In order to fulfill the following condition, the confidence scores have to be normalized:

(1) $$\sum_{c=1}^{C}CS_{c}^{M_{i}}=1;i=1,2,..N$$

The fuzzy rank for a class *c* using $M_{i}$ model is generated by taking the complement of the Gaussian density Function, as shown below:

(2) $$R_{c}^{L_{i}}=\left(1-exp^{-\frac{{\left(CS_{c}^{M_{i}}-1.0\right)}^{2}}{2\times 1.0}}\right),i=1,2,...,N;C=1,2,..,C$$

As a result, it should be noted that $R_{c}^{M_{i}}$ lies between [0, 1] and lowest value is said to be the winner which is analogous to top ranked in conventional ranking.

Let, $K^{M_{i}}$ represents the set of top K fuzzy ranked classes generated by the model $M_{i}$. It is to be noted that $K^{M_{i}}$ and $K^{M_{j}}$ (i≠j) might differ as they belong to two different classifier models. The complement of confidence score sum $CSS_{c}$ and the rank sum $RS_{c}$ relative to a class *c* is determined as follows:

(3) $$CSS_{c}=1-\frac{1}{N}\sum_{i=1}^{N}\left\{\begin{array}{cc} CS_{c}^{M_{i}}=CS_{c}^{M_{i}} & ,ifR_{c}^{M_{i}}\epsilon K^{M_{i}}\\ CS_{c}^{M_{i}}=P_{c}^{CS}\; & ,Otherwise \end{array}\right.$$

(4) $$RS_{c}=\sum_{i=1}^{N}\left\{\begin{array}{cc} R_{c}^{M_{i}}=R_{c}^{M_{i}} & ,ifR_{c}^{M_{i}}\epsilon K^{M_{i}}\\ R_{c}^{M_{i}}=P_{c}^{R}\; & ,Otherwise \end{array}\right.$$

where $P_{c}^{R}$ and $P_{c}^{CS}$ are the penalties, which are assigned to a class *c* if it does not belong to the set of top K ranks (1 in our case). $P_{c}^{R}$ and $P_{c}^{CS}$ are the hyper-parameters and for our case $P_{c}^{R}$ and $P_{c}^{CF}$ have values of 0.33 and 0.05 respectively which is obtained experimentally. These particular set of values have yielded the maximum accuracy score for our dataset. Both these penalties revoke the possibility of class c to likely become a winner. The combination of $CSS_{c}$ and $RS_{c}$ are multiplied to obtain the final score used for the final ranking, which is defined as follows:

(5) $$FS_{c}=RS_{c}\times CFS_{c}$$

Finally, a class with the smallest (minimum) final score is selected as the predicted class of the input sample as shown in the equation below:

(6) $$class\left(X\right)=argmin\left(FS_{c}\right);c=1,2,..,C$$

## 6. Results

In this section, we have provided a discussion about the results obtained from the four base learners, i.e., VGG16, ResNet50, Inception-V3, and Xception. The later part of this section also reports the explainability of the base learners using Grad-Cam and the outcomes observed after applying the proposed FRLF method.

To analyze the obtained outcomes, the metrics and the equations used to compute the values of the metrics have been shown through Equations (7)–(11)

(7) $$A_{c}=\frac{TP+TN}{TP+TN+FP+FN}$$

(8) $$P_{r}=\frac{TP}{TP+FP}$$

(9) $$S_{n}=\frac{TP}{TP+FN}$$

(10) $$S_{p}=\frac{TN}{FP+TN}$$

(11) $$F_{m}=\frac{2\times P_{r}\times S_{n}}{P_{r}+S_{n}}$$

where, *TP*, *TN*, *FP*, and *FN* are defined as follows:

- True Positives (*TP*): True positives are the case when the actual class of the data point was True (1) and the predicted class is also True (1).
- True Negative (*TN*): True negatives are the case when the actual class of the data point was False (0) and the predicted class is also False (0).
- False Positive (*FP*): False positives are the case when the actual class of the data point was False (1) and the predicted class is True (1).
- False Negative (*FN*): False negatives are the case when the actual class of the data point was True (1) and the predicted class is False (0).

### 6.1. Results of Base Learners

After training the base learners, i.e., VGG16, ResNet50, Inception-V3, and Xception, each of them was then evaluated on the test set. Table 3 shows the results obtained by the four base learners.

From Table 2, it is to notice that both VGG16 and Xception models obtained the highest accuracy among all the base learners, attaining an accuracy of 95.34%. Both ResNet50 and InceptionV3 models exhibit the lowest performance by providing an accuracy of 93.04% for the test set. Despite acquiring the same truthfulness in both cases, the models differ in several falsepositives and falsenegatives. The experimental analysis also reveals that Inception-V3 and Xception models predict PD patients more accurately as they misclassify the least number of PD patients. On the other hand, VGG16 and ResNet50 can predict non-PD patients more accurately.

**Figure 8:**
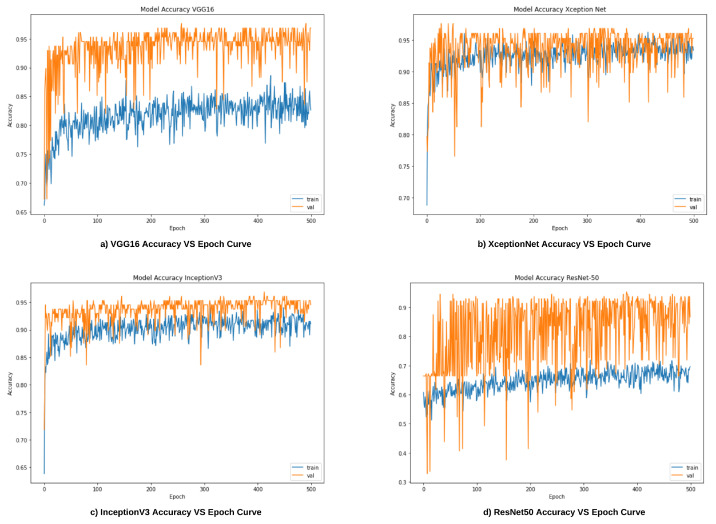
Accuracy vs. Epoch curves for the base learners.

Figure 8a–d illustrates the accuracy vs. epoch curves for the train and test sets for all four base CNN models.

**Figure 9:**
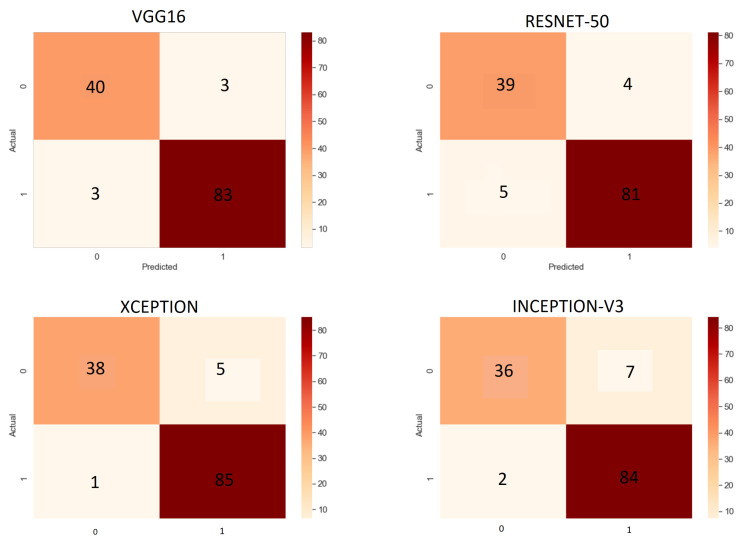
Confusion matrices for all base learners.

Figure 9 depicts the confusion matrix for all the base learners evaluated on the test set.

### 6.2. Grad-Cam Analysis of Base Learners

**Figure 10:**
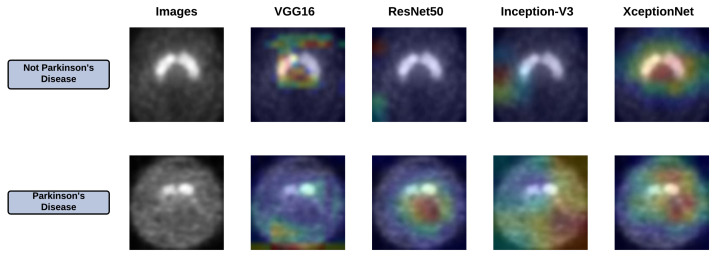
Grad-Cam of all the base learners.

The base learners have facilitated impressive accuracy in the classification of PD and non-PD, yet the biggest problem is in their explainability, which is the vital aspect of understanding and debugging. To understand where the base learners are looking into the input images, we have provided Grad-Cam analysis [45]. This method uses the gradients of a target class, which flows through the final convolutional layer to generate a concentrated map emphasizing the ROI. Figure 10 depicts the ROI obtained by applying Grad-Cam for all the base learners.

### 6.3. Results of Ensemble Approach

After obtaining the results from the base learners, we have ensembled the outcomes using the previously mentioned FRLF method to enhance the overall recognition performance of the proposed system. We have also experimented with a few other basic ensemble techniques like Sum Rule, Product Rule, and Majority Voting to compare the outcomes with the FRLF technique. The working strategy of sum rule, product rule, and majority voting is mentioned here in brief.

Let there be *N* different models ($M_{1}$, $M_{2}$, …, $M_{N}$). Let the *i*th model in consideration be $M_{i}$ whose confidence score are ($CS_{1}^{M_{i}}$, $CS_{2}^{M_{i}}$, …, $CS_{C}^{M_{i}}$) where *C* are the total number of classes in consideration. Then for sum rule [46], the final equation for the prediction of the class for a particular input *X* is defined as:

(12) $$FC_{c}^{Sum\;Rule}=\sum_{i=1}^{N}CF_{c}^{M_{i}},i=1,2,...,N;c=1,2,...,C;$$

(13) $$class\left(X\right)=argmax\left(FC_{c}^{Sum\;Rule}\right),c=1,2,...,C;$$

similarly, for the product rule [47], the equation is deduced as follows:

(14) $$FC_{c}^{Product\;Rule}=\prod_{i=1}^{N}CF_{c}^{M_{i}},i=1,2,...,N;c=1,2,...,C;$$

(15) $$class\left(X\right)=argmax\left(FC_{c}^{Product\;Rule}\right),c=1,2,...,C;$$

for the majority voting [47] ensemble approach, the equation for the final class prediction comes out to be:

(16) $$P_{i}=argmax\left(\left[CS_{1}^{M_{i}},CS_{2}^{M_{i}},\ldots ,CS_{C}^{M_{i}}\right]\right),i=1,2,...,N;$$

(17) $$class\left(X\right)=MaxCount\left(P_{i}\right),i=1,2,...,N;$$

where, $FC_{c}$, $P_{i}$ and MaxCount are the final confidence score for a class *c*, prediction of a model with the highest probability and a function which returns the category which has the highest number of occurrences for a given input *X*.

Table 4 reflects the results obtained after applying these ensemble techniques.

From Table 4, it is to notice that the applied sum rule, product rule, majority voting, and the proposed FRLF method produce an accuracy of 96.89%, 92.25%, 96.12%, and 98.45%. Looking into the accuracy obtained by the ensembled approach, all of them except the product rule performed better than the base learners. Despite achieving the lowest accuracy by-product rule, all the four ensembles can predict the PD patients more accurately by only misclassifying one PD patient as a non-PD patient, i.e., falsenegative, yet they differ in several false positives.

**Figure 11:**
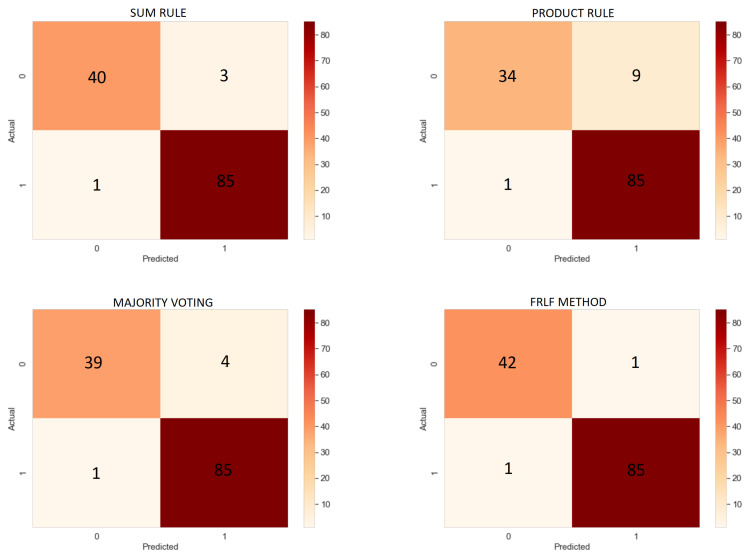
Confusion Matrices of Ensemble method.

The FRLF ensemble-based approach performed significantly higher than the base learners as well as the other ensembled methods. In contrast to supplementary ensemble approaches, the FRLF method misclassified only 2 images, 1 for each of the falsenegatives and falsepositives, obtaining the highest among all the metrics taken into consideration. Figure 11 shows the confusion matrices obtained from all the ensemble approach.

### 6.4. Comparison

Table 5 compares the performance of the proposed FRLF system for the classification of Parkinson’s disease with some past works mentioned in the literature.

Authors in [48] used the dataset that was considerably smaller (19 PD patients and 27 healthy subjects) than the PPMI MRI dataset. They have achieved 86.96% recognition accuracy which is also lesser than our proposed approach. Authors in [19] developed a DL-based model using LIME and VGG16 for the early diagnosis of Parkinson’s disease using the same PPMI dataset and obtained 95.20% accuracy that is relatively lesser than our proposed technique. From the remaining entries of this table, it can be observed that the works mentioned through [12,13,14,20,21] also performed the same task of predicting Parkinson’s disease on the same PPMI dataset. Looking into the obtained accuracies, it can be said that our proposed technique outperforms all the works.

## 7. Software Tool

**Figure 12:**
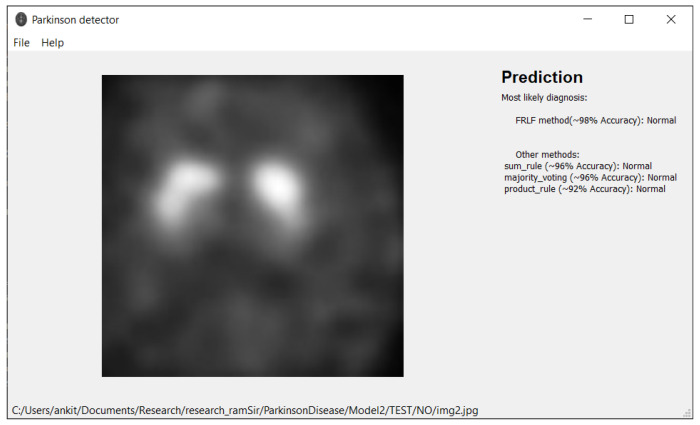
Illustration of prediction made by the software tool while classifying the DaTscan image as “Normal” class.

**Figure 13:**
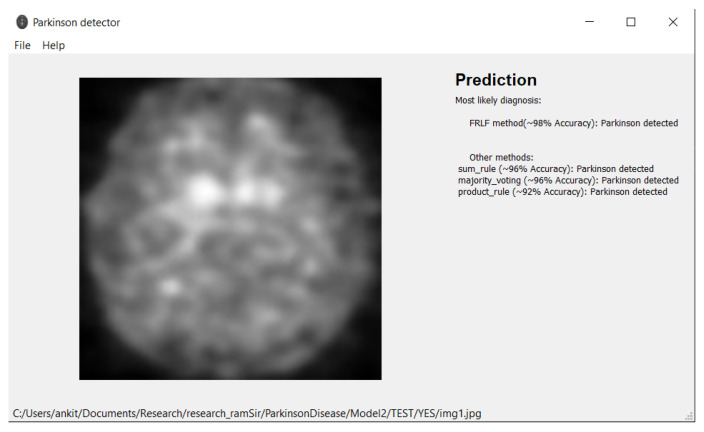
Illustration of prediction made by the software tool while classifying the DaTscan image as “PD” class.

Based on the proposed model, we have developed an application provided in [49] for working with MRI images that can be used by any medical personnel as a support tool for fast preliminary diagnosis. The application is written in Python and runs in both Windows and Linux environment. The user interface is implemented using the Qt library. Our application can work directly with Dicom files (.dcm) from an MRI machine or with any image files (jpg, png, etc.) exported from DICOM viewers. We provided a simple user interface with drag-and-drop support. Figure 12 and Figure 13 depict the outcomes of the application. In the application, we implement all presented ensemble approaches (Sum Rule, Product Rule, Majority voting, and new FRLF method). All ensemble methods use 4 neural networks: VGG-16, ResNet 50, InceptionV3, and Xception. Application requirements include:

- Operating system:
  – Windows 7 or later
  – Ubuntu 16.04 or later
  – Mac OS 10.12.6 (Sierra) or later (64-bit) (no GPU support)
- Python 3.6 or later
- Hard Drive-Maximum 4GB of free space
- Processor-Intel Core i3
- Internet connection-wideband connection for first use (for neural networks downloading)
- Admin privileges are not a requirement.

Note: prediction from the application cannot be used as a medical diagnosis.

## 8. Discussion

Parkinson’s disease has a prevalence rate of 1% in the over-60 age group, and it is the second most common brain disease after Alzheimer’s disease. In addition to assisting practitioners in the process of disease diagnosis, the outcomes of this model will enable them to take timely action before patients’ disorders become more serious.

Based on previously stated results, we can safely comment that our method works effectively on the PPMI dataset, and achieves an accuracy of 98.45%. Also, in the medical image analysis domain especially, it is absolutely necessary to reduce the number of mis-classifications because a false diagnosis can cause physical, emotional and psychological damage to the patient and his/her family. We have observed that the number of false positives and false negatives gets reduced significantly when we have used the FRLF ensemble technique as compared to when we use the Sum Rule, Product Rule and Majority Scoring technique.

One limitation of our work is the number of mis-classifications. Though it is less than most other methods tested on the dataset, we still have wrongly classified images and hence this cannot be used for medical diagnosis with a 100% accuracy. Also, we do not know if the FRLF method is domain-specific or can be applied on other diseases except for Parkinson’s disease. We plan to reduce the number of inaccurate classifications and to test the FRLF ensemble technique on other datasets.

## 9. Conclusions

In the present work, we have proposed an ensemble of DL models to predict Parkinson’s disease effectively using the PPMI DaTscan images. We have designed a fuzzy ensemble model, called FRLF, which is applied on the confidence scores of four classic DL models- VGG16, ResNet50, Inception-V3, and Xception to enhance the overall results of the model. From the results reported in the above section, we can ensure that the proposed model achieves state-of-the-art performance. Recognition accuracy, Precision, Sensitivity, Specificity, F1-score of the proposed model are 98.45%, 98.84%, 98.84%, 97.67%, and 98.84% respectively. We have also incorporated our model in a GUI-based software tool for public use that instantly detects Parkinson’s disease in DaTscan images given to it as inputs. This can play a significant role in detecting Parkinson’s disease in real-time. Our work is primarily based on DaTscan images. We have not yet extended our work to MRI scans or CT scans, which is our plan for future work in this domain. 

## Figures and Tables

**Table 1:** A comparative study of some past methods related to the proposed work.

Work Ref.	Dataset	Method/Classifier	Accuracy	Sensitivity	Specificity
[8]	Custom	SVM on rsfMRI	86.96%	78.95%	92.59%
[9]	PPMI	SVM	93%	93%	92%
[10]	PPMI	Sparse feature selection model	80%	84.70 ± 19.29%	-
[11]	PPMI	PCA followed by SVM	>90%	>90%	>90%
[12]	PPMI	SVM with striatal binding ratio	96.14%	95.74%	77.35%
[13]	PPMI	SVM	92.6%	91.2%	93.1%
[14]	PPMI	ANN	94%	100%	88%
[15]	PPMI	AlexNet	88.9%	-	-
[18]	Custom	CNN	85%	-	-
[19]	PPMI	VGG-16	95.2%	-	90.9%
[20]	PPMI	InceptionV3	98.4%	98.8%	97.6%
[21]	PPMI	AlexNet and LeNet	95±0.3%	-	-
[31]	PD dataset	KNN	98.46%	-	-
[32]	PPMI	SVM with linear kernel classifiers	96%	-	-
[33]	Custom	Modified Grey Wolf Optimization	94.83%	-	-
[34]	Custom	Optimized cuttlefish algorithm	94%	-	-
[35]	PPMI	PCA and ANN	97%	-	-
[36]	Custom	ROI based diagnosis	86.67%	-	-

**Table 2:** Dataset details.

Category	PD	Non-PD	Total
Train	346	170	516
Test	86	43	129

**Table 3:** Observed results from the DL models for the prediction of PD.

Model	Accuracy	Precision	Sensitivity	Specificity	F1-Score
**VGG16**	95.34%	96.51%	96.51%	93.02%	96.51%
**ResNet 50**	93.02%	95.29%	94.19%	90.69%	94.74%
**Inception-V3**	93.02%	92.31%	97.67%	83.72%	94.81%
**Xception**	95.34%	94.44%	98.84%	88.37%	96.59%

**Table 4:** Results obtained after applying different ensemble approaches.

Model	Accuracy (in %)	Precision	Sensitivity	Specificity	F1-Score
Sum Rule	96.89%	96.59%	98.84%	93.02%	97.70%
Product Rule	92.25%	90.42%	98.84%	79.07%	94.44%
Majority Voting	96.12%	95.5%	98.84%	90.69%	97.59%
**FRLF** **method**	**98.45%**	**98.84%**	**98.84%**	**97.67%**	**98.84%**

**Table 5:** Comparison of our proposed method with some past methods found in the literature.

Work Ref.	Method/Classifier	Accuracy	Dataset Used
[12]	SVM with Striatal Binding Ratio	96.14%	PPMI
[13]	PCA with SVM	92.60%	PPMI
[14]	Custom ANN	94.00%	PPMI
[19]	VGG-16	95.20%	PPMI
[20]	InceptionV3	98.40%	PPMI
[21]	LeNet and AlexNet	95% ± 0.30%	PPMI
[48]	t-test and SVM	86.96%	PPMI
**Proposed**	**CNN models + FRLF**	**98.45%**	**PPMI**

## Data Availability

Dataset used here is a public one.
